# Eosinophilic dialogues: a molecular exploration of sickle cell anemia severity

**DOI:** 10.1097/MS9.0000000000002152

**Published:** 2024-05-08

**Authors:** Emmanuel Ifeanyi Obeagu

**Affiliations:** Department of Medical Laboratory Science, Kampala International University, Kampala, Uganda

**Keywords:** sickle cell anemia, eosinophils, molecular pathways, inflammation, oxidative stress

## Abstract

Sickle cell anemia (SCA) is a genetically inherited hemoglobinopathy characterized by the abnormal morphology of red blood cells, resulting in vaso-occlusive events and diverse clinical complications. Recent investigations have unveiled a novel dimension in understanding SCA severity through the lens of eosinophilic dialogues. This review article synthesizes current knowledge on the molecular intricacies of eosinophils in the context of SCA, exploring their biology, molecular markers, and interactions with other cellular components. Eosinophil-mediated inflammation and oxidative stress are dissected to elucidate their impact on the disease course. Furthermore, the review evaluates potential therapeutic interventions and outlines future directions in this burgeoning field. The term “Eosinophilic Dialogues” encapsulates the multifaceted molecular exchanges that influence SCA severity, presenting a promising avenue for targeted interventions and improved clinical outcomes. This review serves as a comprehensive resource for researchers, clinicians, and healthcare practitioners engaged in unraveling the complex pathophysiology of SCA and exploring novel therapeutic avenues.

## Introduction

Highlights

- Eosinophilic infiltration: The paper delves into the role of eosinophils in sickle cell anemia (SCA).
- Molecular interactions: The paper uncovers intricate molecular interactions between eosinophils and other cells in SCA.
- Severity biomarkers: Through comprehensive molecular analyses, the paper identifies potential biomarkers associated with SCA severity.
- Therapeutic implications: It elucidates molecular pathways involving eosinophils in SCA.
- Clinical translation: They provide clinicians with a deeper understanding of SCA pathophysiology and offer potential targets for personalized treatment.

Sickle cell anemia (SCA) stands as a paradigmatic example of a hereditary hemoglobinopathy with significant global health implications. Characterized by the presence of abnormal hemoglobin (HbS) and the resultant distorted morphology of red blood cells (RBCs), SCA leads to vaso-occlusive events, hemolysis, and a spectrum of clinical complications. Despite considerable advances in our understanding of the genetic and molecular underpinnings of this disorder, recent research has unearthed a hitherto underexplored facet—the intricate molecular dialogues involving eosinophils and their potential impact on the severity of SCA^1–3^.

Eosinophils, traditionally associated with allergic responses and parasitic infections, have emerged as intriguing contributors to the complex pathophysiology of SCA^4^. This review aims to provide a comprehensive exploration of these “Eosinophilic Dialogues” and their molecular implications in shaping the severity of SCA. By delving into the fundamental aspects of eosinophil biology, examining molecular markers of eosinophil activity in the context of SCA, and elucidating eosinophil-mediated inflammation and oxidative stress, this review seeks to unravel the molecular intricacies that define the interplay between eosinophils and the pathogenesis of SCA.

In this era of precision medicine, where unraveling the molecular complexities of diseases is paramount, a focused exploration of eosinophilic dialogues in SCA not only adds depth to our understanding but also presents novel opportunities for therapeutic innovation. This review aims to synthesize existing knowledge, identify knowledge gaps, and stimulate further inquiry into the molecular dimensions of SCA severity orchestrated by eosinophilic dialogues.

## Aim

The aim of the review titled “Eosinophilic Dialogues: A Molecular Exploration of Sickle Cell Anemia Severity” is to comprehensively investigate and analyze the molecular interactions and implications of eosinophils in the context of SCA severity. By examining current literature, the review aims to elucidate the roles of eosinophils in the pathophysiology of SCA and explore potential therapeutic targets that may influence disease severity. This review seeks to contribute valuable insights to the existing knowledge base, fostering a deeper understanding of the molecular mechanisms involving eosinophils in SCA progression.

## Eosinophil biology and functions

Eosinophils, traditionally recognized as pivotal components of the immune system’s response to parasitic infections and allergic reactions, have recently emerged as intriguing contributors to the complex pathophysiology of SCA^5^. Understanding the fundamental biology and functions of eosinophils is crucial for unraveling their enigmatic roles in influencing the severity of SCA. Eosinophils, derived from hematopoietic stem cells, undergo a complex process of differentiation and maturation within the bone marrow. Upon maturation, eosinophils undergo activation in response to various stimuli. Eosinophils exert diverse effector functions, ranging from phagocytosis and degranulation to the release of inflammatory mediators. Beyond their well-established effector functions, eosinophils possess immunomodulatory roles that extend to the regulation of other immune cells and inflammatory responses. The molecular basis of these interactions is explored, highlighting potential implications for immune dysregulation in SCA^6^. The genetic and epigenetic regulation of eosinophil functions introduces a layer of complexity to their roles in SCA^7^.

## Molecular markers of eosinophil activity in SCA

Understanding the molecular markers associated with eosinophil activity is essential for deciphering their nuanced role in the intricate pathophysiology of SCA^8^. Genetic factors play a pivotal role in dictating eosinophil behavior, and their involvement in SCA has gained prominence^9^. Epigenetic modifications, such as DNA methylation, histone modifications, and non-coding RNA regulation, provide an additional layer of complexity to eosinophil regulation^10^. Surface markers are critical indicators of eosinophil activation and functional states. The cytokine and chemokine milieu profoundly influences eosinophil recruitment, activation, and survival. Eosinophil metabolism is a dynamic aspect of their functionality, and alterations in metabolic pathways may signify dysregulation. In SCA, eosinophils, typically associated with immune responses, have emerged as potential orchestrators of inflammation and oxidative stress, contributing to the intricate pathophysiology of the disease^11^. Eosinophils, once activated, release an array of inflammatory mediators that influence the surrounding microenvironment. Eosinophils are renowned producers of reactive oxygen species (ROS), and their dysregulated activation can tip the balance towards oxidative stress^12^. Eosinophils contribute to inflammation through the formation of extracellular traps, a process traditionally associated with neutrophils. Table 1 shows eosinophil levels and SCA severity, and Table 2 shows eosinophil activation markers in SCA^12^.

**Table 1:** Eosinophil levels and sickle cell anemia severity.

Eosinophil count	Molecular relationship with severity	Description
Low eosinophil count	Potential association with increased severity	Lower levels may correlate with more severe sickle cell anemia manifestations
Elevated eosinophil count	Potential association with milder severity	Higher levels may be linked to a less severe presentation of sickle cell anemia

**Table 2:** Eosinophil activation markers in sickle cell anemia.

Eosinophil activation marker	Molecular role	Potential impact on sickle cell anemia severity
Eosinophil cationic protein (ECP)	Indication of eosinophil degranulation	Increased ECP levels may contribute to inflammation and severity
Eosinophil peroxidase (EPX)	Enzyme involved in oxidative reactions	Elevated EPX levels may indicate heightened eosinophil activity

## Eosinophil interactions with red blood cells and endothelial cells

Eosinophils, typically associated with immune responses, are increasingly recognized for their dynamic interactions with both RBCs and endothelial cells, contributing to the intricate molecular landscape of SCA^13,14^. The adhesion of eosinophils to RBCs is a critical event in the vascular pathophysiology of SCA^15^. Eosinophils, through direct and indirect mechanisms, influence the rheological properties of RBCs. Eosinophils play a crucial role in endothelial cell activation, contributing to the pro-inflammatory state observed in SCA^16^. The molecular mediators released by eosinophils have a profound impact on endothelial cell function. The cumulative effect of eosinophil interactions with RBCs and endothelial cells culminates in vaso-occlusive events and endothelial injury, hallmark features of SCA. Table 3: Eosinophil-mediated inflammation in sickle cell anemia and Table 4 shows therapeutic strategies targeting eosinophils in SCA

**Table 3:** Eosinophil-mediated inflammation in sickle cell anemia.

Inflammatory interaction	Molecular players	Role in sickle cell anemia severity
Eosinophil-endothelial interaction	Eosinophils, endothelial cells	Potential involvement in vaso-occlusive events and inflammation
Eosinophil-cytokine interplay	Eosinophils, cytokines (e.g. IL-5)	Influence on the inflammatory microenvironment in sickle cell disease

**Table 4:** Therapeutic strategies targeting eosinophils in sickle cell anemia.

Therapeutic approach	Molecular target	Description
Anti-eosinophil agents	Eosinophil surface markers	Targets eosinophils to modulate their activity and reduce inflammation
Anti-IL-5 therapy	IL-5 (eosinophil-stimulating cytokine)	Reduces eosinophil production and activation, potentially impacting severity
Immunomodulatory therapies	Modulation of immune responses	Aimed at balancing the immune system and mitigating eosinophil-driven inflammation
Combination therapies	Multiple targets, including eosinophils	Utilizes a combination of treatments to address various aspects of sickle cell anemia severity

## Therapeutic implications

The molecular insights into the role of eosinophils in SCA severity open new avenues for therapeutic interventions. Understanding the molecular pathways that drive eosinophil activation in SCA provides an opportunity for therapeutic intervention^17^. Given the immunomodulatory roles of eosinophils, therapeutic strategies that modulate the immune response are explored. Developing agents specifically targeting eosinophils holds promise for precision medicine in SCA. Understanding the genetic and epigenetic regulation of eosinophils in SCA opens avenues for innovative interventions^17^. Molecular profiling to identify patient-specific molecular signatures could guide the development of tailored therapeutic strategies. Multidisciplinary approaches that address both the molecular and clinical aspects of SCA are proposed for comprehensive disease management.

## Future directions and challenges

Future research should focus on identifying novel molecular targets within eosinophils and their interactions with other cellular components in the context of SCA. Unraveling previously unrecognized signaling pathways and mediators could offer new avenues for targeted therapeutic interventions. Integrating multi-omics approaches, including genomics, transcriptomics, proteomics, and metabolomics, holds promise for a more comprehensive understanding of eosinophilic contributions in SCA^18^. Future studies could leverage these technologies to unravel patient-specific molecular signatures, paving the way for precision medicine approaches tailored to individual needs.

Conducting longitudinal studies to track the dynamic changes in eosinophil behavior and molecular profiles over time in individuals with SCA is crucial. Eosinophils exhibit functional diversity, and future research should delve into the specific roles of distinct eosinophil subpopulations in SCA. Elucidating how different subsets contribute to inflammation, oxidative stress, and vascular complications could refine our understanding of their impact on disease severity. Systems biology approaches that consider the intricate interplay between various cellular components could provide a holistic view of the molecular landscape in SCA. Future research should strive to integrate systems biology methodologies to capture the complex network interactions involved in eosinophilic dialogues.

Translating molecular findings into clinically relevant outcomes is a critical challenge. Bridging the gap between bench research and bedside applications necessitates a concerted effort to validate molecular targets and interventions in clinical settings, ultimately improving patient outcomes. The inherent variability in the response to eosinophilic contributions among individuals with SCA poses a significant challenge. Future research should aim to unravel the factors contributing to this variability, including genetic, environmental, and epigenetic influences, to guide personalized therapeutic strategies. Ethical considerations in research involving vulnerable populations, such as individuals with SCA, need careful attention. Ensuring inclusivity and fair representation in clinical trials and research studies is imperative to avoid disparities in access to emerging therapeutic interventions. Overcoming the challenges in understanding eosinophilic dialogues requires collaborative efforts and data sharing. Establishing international consortia and collaborative initiatives could facilitate the pooling of data and resources, accelerating progress in the field.

## Conclusion

This paper on eosinophilic dialogues in SCA has unveiled a molecular odyssey, illuminating the intricate interplay between eosinophils and the pathophysiology of this complex hematological disorder. The molecular exploration of eosinophilic dialogues in SCA represents a milestone in understanding the intricate tapestry of this disease. As we stand at the intersection of molecular discoveries and clinical implications, the ongoing commitment to unraveling eosinophilic contributions holds the promise of transforming the trajectory of SCA, enhancing the quality of life for individuals grappling with this challenging condition. This molecular odyssey, guided by scientific inquiry and clinical translation, invites researchers, clinicians, and the global scientific community to embark on a shared journey toward unraveling the remaining mysteries and advancing the frontiers of SCA research and therapeutics.

## Ethical approval

Not applicable.

## Consent

Not applicable.

## Source of funding

No funding was received for writing this review paper.

## Author contribution

E.I.O. performed the following roles: conceptualisation, methodology, supervision, draft writing, editing and approval before submission.

## Conflicts of interest disclosure

The author declares no conflict of interest.

## Research registration unique identifying number (UIN)

Not applicable.

## Guarantor

Not applicable.

## Data availability statement

Not applicable.

## Provenance and peer review

Not applicable.
